# Validation of fNIRS measurement of executive demand during walking with and without dual-task in younger and older adults and people with Parkinson’s disease

**DOI:** 10.1016/j.nicl.2024.103637

**Published:** 2024-07-01

**Authors:** Alexander Kvist, Lucian Bezuidenhout, Hanna Johansson, Franziska Albrecht, David Moulaee Conradsson, Erika Franzén

**Affiliations:** a Department of Neurobiology, Care Sciences and Society, Division of Physiotherapy, Karolinska Institutet, Stockholm, Sweden; b Department of Health and Rehabilitation Sciences, Division of Physiotherapy, Stellenbosch University, Cape Town, South Africa; c Women’s Health and Allied Health Professionals Theme, Medical Unit Occupational Therapy & Physiotherapy, Karolinska University Hospital, Stockholm, Sweden; d Stockholm Sjukhem Foundation, Mariebergsgatan 22, 112 19 Stockholm, Sweden

**Keywords:** Gait, Parkinson, fNIRS, Dual-task walking, Validation

## Abstract

**Background:**

Walking with a concurrent cognitive task (dual-task walking) can pose a challenge to some populations due to aging or neurodegenerative disease. These tasks require cognitive resources involving the prefrontal cortex and can be studied using functional near-infrared spectroscopy (fNIRS). An important step in understanding fNIRS measures during such walking tasks is validating that measures reflect the demands of the tasks and not confounding sources or movement artifacts.

**Aim:**

This study aimed to investigate the validity of fNIRS measures of prefrontal cortex activity as an indicator of executive demand during usual walking (single-task) and dual-task walking against clinical and objective measures of motor behavior in young adults, older adults, and people with Parkinson’s disease (PD), by evaluating several validation hypotheses.

**Methods:**

In total, 133 participants were recruited from younger adults (18–50 years, n = 42), older adults (≥60 years, n = 49) and people with PD (≥60 years, n = 42). Activity in the prefrontal cortex during walking with and without an auditory Stroop task was measured with fNIRS. A combined hemoglobin measure (correlation-based signal improvement, CBSI) was calculated for use in a region of interest analysis in the dorsolateral prefrontal cortex (dlPFC). Pre-registered hypotheses regarding convergent validity, discriminant validity and known group validity were tested. An exploratory analysis of different hemoglobin measures was also performed.

**Results:**

Increases in dlPFC activity were found from single- to dual-task walking in the younger adults group and from rest to single-task walking in the older adults and PD groups. In line with hypotheses, a positive relationship was found between between dlPFC activity during dual-task walking and dual-task cost in the younger adults group, as well as a positive relationship to step time variability during single-task walking and a negative relationship to walking speed during single-task walking in the PD group. However, several clinical and gait measures lacked a relationship with dlPFC activity.

**Conclusion:**

The fNIRS results point towards the CBSI measure of dlPFC activity being a valid measure of executive demand during both single and dual-task walking. Some relationships between clinical and gait measures and brain activity during walking need further investigation.

## Highlights

• The validity of fNIRS measurement of executive demand during walking with and without a dual-task was tested via validation hypotheses relating to convergent, discriminant and known-group validity.
• Relationships between activity in the dorsolateral prefrontal cortex and dual-task cost, step time variability and walking speed was found in accordance with validation hypotheses.
• A combined hemoglobin measure, correlation-based signal improvement (CBSI), was considered a valid measure of executive demand during walking with and without dual-task.

## 1 Introduction

Walking in everyday life involves many complex walking tasks such as turning (Glaister et al., 2007), walking while talking or performing a simultaneous cognitive task (i.e. dual-task) or navigating around obstacles (Galna et al., 2009). Such complex walking tasks require both motor and cognitive skills including amongst others balance, motor planning and attention (Clark, 2015). These skills are subject to decline due to aging (Papegaaij et al., 2014, Seidler et al., 2010) or neurodegenerative disease (McIsaac et al., 2018). Studies have pointed to a greater involvement of cognitive resources, possibly compensatory, during postural tasks in older adults (Boisgontier et al., 2013) and a distinct difficulty in performing dual-tasks in populations with neurodegenerative disease such as Parkinson’s disease (PD) (Raffegeau et al., 2019). While interference between cognitive and motor tasks during dual-tasking is not fully understood (Ruthruff et al., 2006, Strobach, 2020), neuroimaging could further enhance understanding of the phenomenon during such walking tasks (Clark, 2015).

Walking tasks have been investigated using many neuroimaging modalities such as imagined walking in functional magnetic resonance imaging (fMRI), as well as actual walking with positron emission tomography (PET) and electroencephalography (EEG) (Hamacher et al., 2015). An especially suitable neuroimaging modality for walking tasks is functional near-infrared spectroscopy (fNIRS) due to its mobility and spatial localization ability (Herold et al., 2017). Functional near-infrared spectroscopy provides indirect measurements of brain activity by transmitting near-infrared light through an examined brain structure and utilizing the different optical properties of oxygenated hemoglobin (HbO2) and deoxygenated hemoglobin (HHb) to obtain a measure of the hemodynamic response of the brain (Scholkmann et al., 2014). Due to fNIRS being noninvasive and portable, it can provide measurements during real walking, providing the method with ecological validity as compared to imagined walking. Despite best practice guidelines advocating for reporting both HbO2 and HHb measures (Yücel et al., 2021) in fNIRS studies, there is a recurring argument in favor of combined measures such as hemoglobin difference, total hemoglobin, or more complex measures (Hakim et al., 2022). This is because they can incorporate information from both hemoglobin sources in a single analysis framework (Hakim et al., 2022). One such measure is the correlation-based signal improvement (CBSI) measure, reflecting that HbO2 and HHb should be anti-correlated during functional activation of the brain (Cui et al., 2010).

An important step in understanding fNIRS measures during walking paradigms is to validate that measured brain activity arises as a response to the demands of the walking tasks and not extra-cortical sources, movement artifacts, or other confounding sources. During walking with a dual-task, these demands relate to executive function which involves the dorsolateral prefrontal cortex (dlPFC) and the cingulate cortex (Yogev-Seligmann et al., 2008). Imaging studies have indicated that overground walking generally results in a higher PFC activation compared to resting conditions for older adults and people with neurodegenerative disorders (Kahya et al., 2019, Pelicioni et al., 2019, Stuart et al., 2018, Vitorio et al., 2017). These effects were more prominent during dual-task (Pelicioni et al., 2019, Stuart et al., 2018). There are still only a few studies involving people with PD. These generally indicate a higher prefrontal activity during dual-task walking (Pelicioni et al., 2019) and only sometimes look into more detailed relationships with other correlates such as gait variables and balance (Al-Yahya et al., 2019, Maidan et al., 2016, Mirelman et al., 2017, Vitorio et al., 2020).

Therefore, the aim of this study is to investigate the validity of fNIRS measures of dlPFC activity as an indicator of executive demand during single-task walking and dual-task walking against clinical and objective measures of motor behavior in younger adults, older adults, and people with PD. Hypotheses regarding convergent validity and discriminant validity are tested (de Vet et al., 2011), supposing relationships between dlPFC activity and measures such as step time variability. For example, we expect participants with a higher prefrontal activity during walking to have a higher step time variability, since we expect a greater involvement of cognitive resources during walking to be reflected in increased gait variability. Hypotheses regarding known group validity are also tested, supposing differences in dlPFC activity between disease severity groups and participant groups. All validation hypotheses are listed in Table 1. An exploratory analysis of different hemoglobin measures is also performed, with the expectation that the CBSI measure satisfies the most amount of validation hypotheses.

**Table 1:** Validation hypotheses.

Hypothesis ID	Validity type	Hypothesis	Groups
H1	Convergent	There is an increase in dlPFC activity during single-task walking compared to rest (standing still)	OA, PD
H2	Known-group	The increase in dlPFC activity during single-task walking compared to rest is larger in Hoehn & Yahr (HY) stage 3 & 4 compared to 1 & 2	PD
H3	Convergent	There is an increase in dlPFC activity during dual-task walking (with auditory Stroop) compared to single-task walking	YA, OA, PD
H4	Known-group	The increase in H3 is larger in OA and PD compared to YA	YA, OA, PD
H5	Convergent	A lower balance ability (Mini-BESTest score) should reflect in a higher dlPFC activity during single-task walking (negative interaction effect)	OA, PD
H6	Convergent	A higher (more severe) motor score from MDS-UPDRS part 3 should reflect in a higher dlPFC activity during single-task walking (positive interaction effect)	PD
H7	Convergent	A higher self-perceived walking difficulty (Walk-12 score) should reflect in a higher dlPFC activity during single-task walking (positive interaction effect)	OA, PD
H8	Convergent	A higher step time variability should reflect in a higher dlPFC activity during single-task walking (positive interaction effect)	OA, PD
H9	Convergent	A lower walking speed should reflect in a higher dlPFC activity during single-task walking (negative interaction effect)	OA, PD
*H*10	Convergent	A higher dual-task cost on walking speed should reflect in a higher dlPFC activity during dual-task walking (positive interaction effect)	YA, OA, PD
*H*11	Discriminant	Anxiety (HADS score) should not affect dlPFC activity (no interaction effect)	YA

Abbreviations: YA younger adults, OA older adults, PD Parkinson’s disease, dlPFC dorsolateral prefrontal cortex, H hypothesis, HADS Hospital Anxiety and Depression Scale, MDS-UPDRS Movement Disorder Society-Sponsored Revision of the Unified Parkinson's Disease Rating Scale.

## 2 Material and methods

### 2.1 Participants

The validation study involved participants from three groups: younger adults (N = 42, mean age = 36.0, age SD = 10.1), older adults (N = 49, mean age = 69.3, age SD = 6.7) and people with PD (clinical diagnosis ≥ 6 months before enrolment, N = 42, mean age = 69.3, age SD = 7.4) able to walk without a mobility device for more than 5 min continuously. Participants were recruited through advertisements. Participants from the PD group were measured while on their usual medication schedule.

Exclusion criteria for all groups were speech difficulties, cognitive difficulties affecting the ability to understand and/or follow verbal/written instructions, severe freezing of gait, severe hearing problems, severe visual impairments, or other neurological diseases or conditions that could affect gait and balance. These criteria were assessed through a telephone interview and at the visit, however no specific cutoffs based on cognitive or clinical tests were used to exclude participants.

The study was approved by the Swedish Ethical Review Authority (Dnr 2020–05315 and 2021–01329). Participants received verbal and written information about the study and gave written consent prior to study participation.

### 2.2 Experimental procedure

Experiments took place at the uMOVE core facility, Karolinska University Hospital, Solna, Stockholm. For the older adult and PD groups, the experiment took place across two sessions, while the younger adult group performed the experiment in one session.

During the experimental sessions, clinical tests of balance, disease severity and a neuropsychological test battery were performed, along with fNIRS measurement during a block-based complex walking protocol. After the experiment, questionnaires regarding health status and various personal factors were filled out by participants. A selection of the assessments performed are used for this validation study; the full dataset is detailed in (Franzén, 2023). The older adult and PD groups performed the clinical tests (with disease severity tests only for the PD group) and neuropsychological tests during one session and the fNIRS measurement during another. Questionnaires were filled out digitally or on paper by the participants at home after the first session. The sessions were generally within 2 weeks of each other. Each session took approximately 1.5 – 2 h when performed separately.

Clinical tests of balance were performed for the older adult and PD groups with the Mini-Balance Evaluation Systems Test (Mini-BESTest) (Di Carlo et al., 2016) and disease severity for the PD group with the Movement Disorder Society-sponsored revision of the Unified Parkinson's Disease Rating Scale (MDS-UPDRS) (Goetz et al., 2008). The questionnaires used in this study were the Hospital Anxiety and Depression Scale (HADS) (Zigmond and Snaith, 1983) and the Walk-12 scale for self-assessed walking difficulty (Holland et al., 2006).

### 2.3 fNIRS measurement

The complex walking protocol used for this study previously detailed in (Kvist et al., 2023) contained blocks of different task conditions: straight single-task (ST) walking, standing still while performing an auditory Stroop single-task, and walking while performing an auditory Stroop dual-task (DT). Stimulus duration for each condition was 20 s, followed by approximately 15 s of quiet standing as a rest period (i.e., baseline to compare conditions to). Each condition was performed 6 times. Walking was done at a self-selected speed back and forth along a 30 m long straight path marked by cones in an open lab space. Instructions and the auditory Stroop task were given via a wireless headset and responses were recorded via a microphone on the headset using the Audacity version 3.1.3 software. The auditory Stroop task consisted of the words high and low presented in Swedish, in a congruent or incongruent high or low tone of voice, with the participant being asked to indicate the tone. During the experiment, spatiotemporal gait parameters were captured using the Mobility Lab^TM^ software via three inertial measurement units (Opal, APDM Inc) placed over the lumbar region and both feet.

**Fig. 1:**
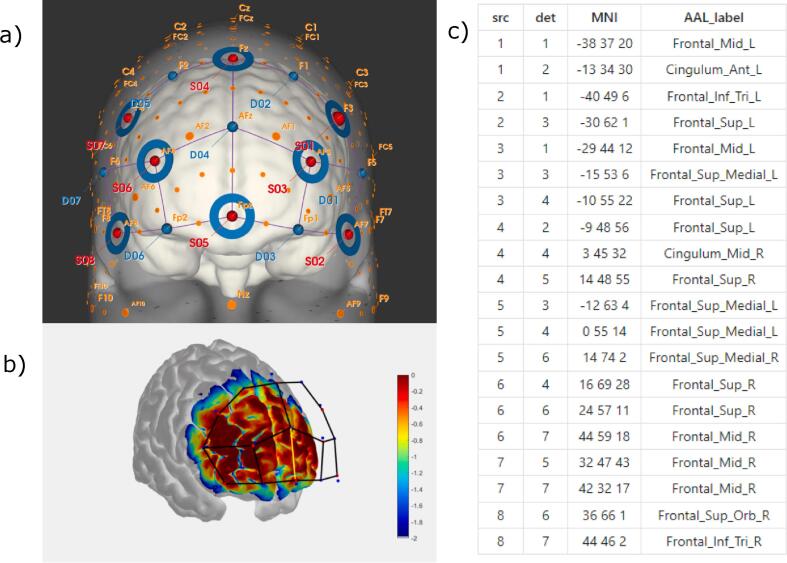
Montage used in the fNIRS experiment. (a): Selected 10–20 positions for optodes (created with NIRsite, NIRx). (b): Sensitivity profile of used montage, created with AtlasViewer. (c): Channel locations in terms of closest Montreal Neurological Institute (MNI) coordinate and closest Automated Anatomical Labeling (AAL) atlas label, also via AtlasViewer.

The fNIRS system used was a NIRSport2 (NIRx) with 8 sources and 8 detectors, with short-separation detection channels for each source to allow for removing superficial blood flow changes in the signal. The optodes transmitted light at 760 and 850 nm, and the sampling frequency was 10 Hz. Data was captured using Aurora (NIRx) (v.1.4). The optodes were fitted to a cap according to the international 10–20 system and placed over the prefrontal area (Fig. 1).

### 2.4 Validation procedure

Validation hypotheses were set up based on current literature on brain activity and cognitive resource use during motor and cognitive tasks (Bakker et al., 2008, Boisgontier et al., 2013, Hamacher et al., 2015, la Fougère et al., 2010, Zwergal et al., 2012), as well as earlier studies on PFC and dlPFC activity during real walking (Bishnoi et al., 2021, Kahya et al., 2019, Kim and Fraser, 2022, Pelicioni et al., 2019, Stuart et al., 2018, Vitorio et al., 2017). The validation hypotheses are presented in Table 1. Our expectation was that the hypotheses would most likely be reflected in the CBSI (Cui et al., 2010) combined measure. Other measures (HbO2, HHb, hemoglobin difference, total hemoglobin) were investigated in an exploratory analysis, using the same tests.

A power analysis of a pilot dataset (https://osf.io/uqy6d) estimated that main condition effects would require 40 to 50 subjects to be well-powered, while interaction effects with continuous covariates, although difficult to estimate, could require further subjects.

### 2.5 Data analysis

Demographic data was compared between groups using R (v4.2.2) (R Core Team, 2022) and the *arsenal* (v3.6.3) and *ggstatsplot* (v0.12.3) packages. Normality was assessed with the Shapiro-Wilk normality test and visually with q-q plots. Comparison was done with the Kruska-Wallis test and post-hoc tests using the Dunn test (where data belonged to 3 groups), or the Mann-Whitney *U* test (where data belonged to 2 groups).

For quality control of fNIRS data, the scalp-coupling index (SCI) as well as peak spectral power (Hernandez et al., 2020, Pollonini et al., 2016) of the fNIRS signal were calculated using the MNE-NIRS Python library (Gramfort et al., 2013, Luke et al., 2021) (v0.6) and the QT-NIRS (Hernandez et al., 2020) MATLAB package (commit 23d5d67), respectively. The peak spectral power calculation used time windows of 5 s. Channels with bad quality data (SCI < 0.7) were replaced with null data (Klein et al., 2022).

Analysis of fNIRS data was performed in the MATLAB NIRS AnalyzIR toolbox (Santosa et al., 2018) (forked version; see Data availability for details). The raw optical density fNIRS data was converted into Δ HbO2 and Δ HHb using the modified Beer-Lambert law (Delpy et al., 1988) with the differential path-length factor (DPF) dependent on age (Scholkmann and Wolf, 2013, Scholkmann et al., 2013). In addition, three combined hemoglobin measures were calculated: total hemoglobin (HbT = HbO2 + HHb), hemoglobin difference (HbD = HbO2 − HHb), and the correlation-based signal improvement (CBSI) signal (Cui et al., 2010) as:

$$\mathrm{CBSI}=\frac{1}{2}\left(\Delta HbO_{2}-\alpha \Delta HHb\right)$$

The first level (subject level) analysis employed a general linear model (GLM) on each hemoglobin type, using pre-whitening and an autoregressive model (AR-IRLS) (Barker et al., 2013) to reduce systemic physiology and motion-induced artifacts. Short-separation channels were used as regressors to further filter out physiological noise. A canonical hemodynamic response function (HRF) was assumed.

The second level (group level) analysis to investigate condition effects and effects of covariates (e.g., step time variability) was done using group-level mixed effects models as detailed in (Santosa et al., 2018). These models utilized first level β variables, representing the change in hemoglobin concentration compared to baseline, along with walking condition as a factor with three levels (one for each condition), covariates, and a random intercept for each subject.

For studying walking condition effects, the mixed model used was:

$$\beta ∼-1+condition+\left(1|Subject\right)$$

For studying the effects of covariates on brain activity during different conditions, the mixed models used for each covariate were:

$$\beta ∼-1+condition+condition:covariate+\left(1|Subject\right)$$

Finally, to reach a single T-statistic for each effect of interest, region of interest (ROI) averaging was performed over the dlPFC, considered as Brodmann area 9 and 46 (Petrides, 2005). Channels in the ROI were selected according to the fNIRS Optodes’ Location Decider (fOLD) toolbox (Zimeo Morais et al., 2018) (v2.2) using the Brodmann atlas and channels were weighted with corresponding specificity to underlying areas.

To account for multiple comparisons of multiple channels and conditions, Benjamini–Hochberg (Benjamini and Hochberg, 1995) false-discovery rate (FDR) corrected p-values were used (q-values) for each model. Significant values were considered as FDR-corrected threshold q < 0.05.

For analyzing gait variables, raw inertial measurement unit data during each block was processed using a strapdown integration 3D position estimation method (https://github.com/alkvi/python-imu-gait-evaluation/tree/phd_study_2) validated in (Kvist et al., 2024). Step time variability was calculated based on the standard deviation of left and right steps according to (Galna et al., 2013). Dual-task cost on walking speed was calculated as:

$$\mathrm{DT}\;cost=-\frac{\mathrm{dual}\;task\;speed-single\;task\;speed}{\mathrm{single}\;task\;speed}*100\%$$

Outliers in gait variables due to unexpected behavior in the protocol (e.g., stopping during a walking block) were excluded from analysis. For visualization purposes, first level β variables from the fNIRS analysis were ROI-averaged and plotted against gait variables with R (v4.2.2) (R Core Team, 2022) using *ggplot2* (Wickham, 2016) and the *lm* function, with Spearman (for robustness to outliers) correlation values added with *stat_cor*.

### 2.6 Missing data

Two subjects from the older adults group were excluded from the fNIRS analysis due to not following the walking protocol, and three subjects from the PD group due to data loss, not following the walking protocol, or not being able to complete the walking protocol. In the older adults group, 7 participants had missing data for the balance score (Mini-BESTest) and 10 participants had no self-reported walking difficulty score (Walk-12). Two participants from the PD group lacked the disease severity score (MDS-UPDRS) and self-reported walking difficulty (Walk-12). HADS data was missing for one participant in the younger adults group. Participants with missing data were not used in the corresponding mixed effects model, and the final number of participants for each effect is reported.

## 3 Results

### 3.1 Participants

In total, 133 subjects (younger adults YA = 42, older adults OA = 49, PD = 42) took part in this validation study (Table 2). The mean age of the YA group was 36.0 years, while the mean age of the OA and PD group was 69.3 years. The PD group was primarily concentrated to Hoehn & Yahr stage II (n = 21) and III (n = 16). The PD group had a worse balance (Mini-BESTest score) and more self-reported walking difficulties (Walk-12 score) than the OA group.

**Table 2:** Participant characteristics.

Demographic measure	YA	OA	PD	Comparison
Number of subjects, n	42	49	42	
Sex, female n (%)	19 (45.3)	21 (42.9)	19 (45.2)	p = 0.97
Age (years), mean (SD), range	36.0 (10.1), 18–50	69.3 (6.7), 60–85	69.3 (7.4), 60–91	**p_YA-OA_ < 0.01**^a^ **p_YA-PD_ < 0.01**^a^ p_OA-PD_ = 1.0^a^
Education (years), mean (SD), range	16.0 (3.1), 11–25	15.1 (2.6), 9–20	15.7 (2.3), 9–20	p = 0.25
Height (cm), mean (SD)	172.4 (10.5)	173.7 (10.2)	173.4 (9.5)	p = 0.79
Weight (kg), mean (SD)	73.2 (14.0)	74.5 (14.0)	75.8 (17.1)	p = 0.80
Balance (Mini-BESTest score), mean (SD), range	N/A	25.3 (2.6), 17–28	23.2 (3.7), 8–28	**p < 0.01**^b^
Walking difficulty (Walk-12 score), mean (SD), range	N/A	1.6 (2.2), 0–7	7.5 (6.4), 0–23	**p < 0.01**^b^
Hoehn & Yahr stage (I-IV)	N/A	N/A	I: 2, II: 21, III: 16, IV: 1	

Abbreviations: YA: younger adults, OA: older adults, PD: Parkinson’s disease, SD: standard deviation.

a Dunn test,

b Mann-Whitney *U* test.

### 3.2 Signal quality

The signal quality in terms of SCI was generally high (around 0.967) during each protocol (supplementary Table S1), reflecting a good optode coupling to the scalp. In terms of peak spectral power (supplementary Table S1), standing while performing the Auditory stroop had the highest power (0.248), reflecting the least amount of movement artifacts, while dual-task walking had the lowest power (0.194). In all conditions, the average peak spectral power was above the customary threshold (Pollonini et al., 2016) for a good signal (0.1).

### 3.3 Convergent and discriminant validity

**Fig. 2:**
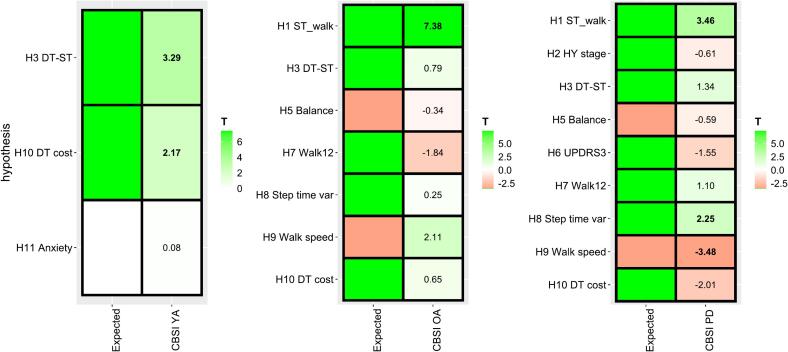
Hypotheses H1-*H*11 visualized for the correlation-based signal improvement (CBSI) measure. In each grid, the color in the left column (Expected) represents the expected direction of an effect according to the hypothesis. The right column of each grid represents the T value of the mixed effects model outcome for the hypothesis. Significant values (q < 0.05) are indicated in bold. Abbreviations: ST single-task, DT dual-task, YA younger adults, OA older adults, PD Parkinson’s disease, HY Hoehn & Yahr.

In the YA group, there was a significant increase in dlPFC activity from single-task walking to dual-task walking (Table 3) (β = 0.34, T = 3.29, q < 0.01) and a positive relationship between dlPFC activity during dual-task walking and dual-task cost (β = 2.76, T = 2.17, q = 0.04), as hypothesized (Fig. 2). There was no significant relationship to anxiety in accordance with hypothesized discriminant validity.

**Table 3:** Results of mixed-effects models for testing hypotheses H1-*H*11 using the CBSI hemoglobin measure. Minus (−) denotes a contrast and a colon (:) denotes an interaction effect.

Hypothesis	Group	Effect	Beta	SE	T	p	q	Included n
H1	OA	ST walking	0.60	0.08	7.38	0.00	0.00	47
H1	PD	ST walking	0.29	0.08	3.46	0.00	0.00	39
H2	PD	HY3/4 − HY1/2	−0.11	0.17	−0.61	0.55	0.55	37
H3	YA	DT walking – ST walking	0.34	0.10	3.29	0.00	0.00	42
H3	OA	DT walking – ST walking	0.06	0.08	0.79	0.43	0.43	47
H3	PD	DT walking – ST walking	0.10	0.08	1.34	0.18	0.18	39
H5	OA	ST walking: balance	−2.34	6.88	−0.34	0.73	0.88	40
H5	PD	ST walking: balance	−3.15	5.32	−0.59	0.56	0.75	37
H6	PD	ST walking: MDS-UPDRS 3 motor score	−2.37	1.52	−1.56	0.12	0.15	37
H7	OA	ST walking: Walk12	−4.57	2.49	−1.84	0.07	0.14	37
H7	PD	ST walking: Walk12	0.93	0.84	1.10	0.27	0.33	37
H8	OA	ST walking: step time variability	0.44	1.74	0.25	0.80	0.80	46
H8	PD	ST walking: step time variability	2.03	0.90	2.25	0.03	0.04	39
H9	OA	ST walking: walking speed	9.79	4.63	2.11	0.04	0.07	46
H9	PD	ST walking: walking speed	–23.78	6.84	−3.48	0.00	0.00	39
*H*10	YA	DT walking: DT walking speed cost	2.76	1.27	2.17	0.03	0.04	41
*H*10	OA	DT walking: DT walking speed cost	0.60	0.91	0.65	0.51	0.51	46
*H*10	PD	DT walking: DT walking speed cost	−2.10	1.05	−2.01	0.05	0.06	39
*H*11	YA	anxiety	0.17	2.05	0.08	0.94	0.94	41

Abbreviations: H hypothesis, YA younger adults, OA older adults, PD Parkinson’s disease, SE standard error, CBSI correlation-based signal improvement, ST single-task, DT dual-task, HY Hoehn & Yahr.

In the OA group, the only significant effect among tested hypotheses was an increase in dlPFC activity during single-task walking compared to the rest condition (β = 0.60, T = 7.38, q < 0.01). The increase was in accordance with hypothesis H1, but the other expected relationships remained non-significant.

In the PD group, there was a significant increase in dlPFC activity during single-task walking (β = 0.29, T = 3.46, q < 0.01), and significant interaction effects between dlPFC activity and walking speed (β = –23.78, T = -3.48, q < 0.01) and step time variability (β = 2.03, T = 2.25, q = 0.04). These interactions were according to the validation hypotheses: a lower walking speed and a higher step time variability were significantly associated with higher brain activity.

**Fig. 3:**
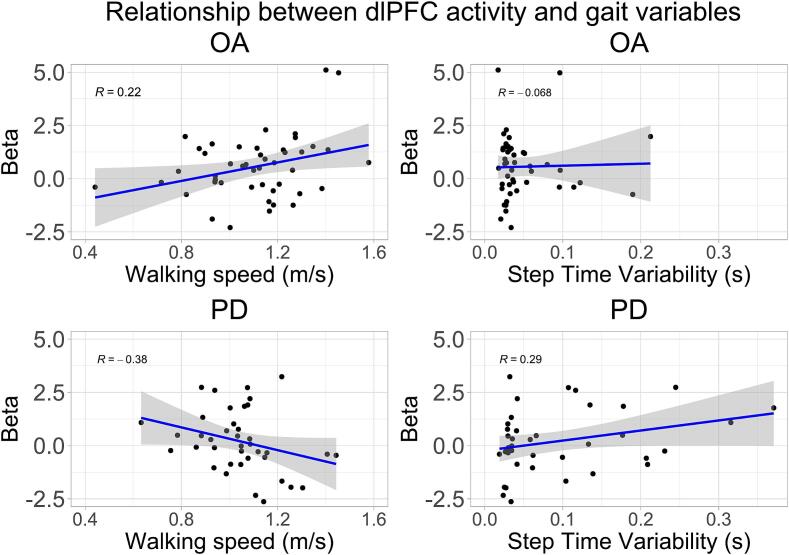
Relationship between subject-level region of interest dlPFC activity and walking speed and step time variability for the OA group (top) and the PD group (bottom). Abbreviations: OA older adults, PD Parkinson’s disease, dlPFC dorsolateral prefrontal cortex.

A regression line through the first level dlPFC activity (β variables) plotted against gait measures for the OA and PD group (Fig. 3) further illustrates the relationship between brain activity and walking performance, showing larger correlation values for the PD group than the OA group.

### 3.4 Known-group validity

None of the hypothesized known-group relationships (hypotheses H2 and H4) held as expected. Contrasting the dlPFC activity during single-task walking for people with PD with mild (Hoehn & Yahr I/II) to moderate/severe disease (III /IV) for hypothesis H2 showed no difference (β = -0.11, T = -0.61, q = 0.55). The dual-task to single-task contrast was not larger in the OA or PD groups compared to the YA group, as hypothesized in H4.

### 3.5 Exploratory analysis of hemoglobin measures

**Fig. 4:**
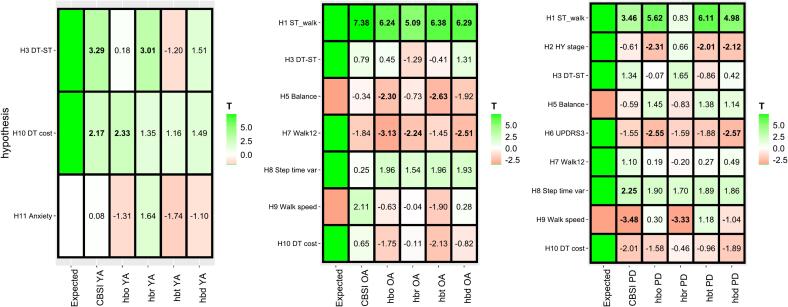
Hypotheses H1-*H*11 visualized for all hemoglobin measures (CBSI, HbO2, HHb, HbT, HbD). For the HHb measure (hbr in the figure), the T values have been modified to have the opposite sign, since an increase in brain activity is reflected in a decrease of HHb. Abbreviations: ST single-task, DT dual-task, YA younger adults, OA older adults, PD Parkinson’s disease, HY Hoehn & Yahr.

The exploratory analysis generally showed a consistent direction of effect across the different hemoglobin measures, especially for significant effects such as single-task walking in hypothesis H1 (Fig. 4). The CBSI measure had the greatest number of significant effects in line with hypotheses. For the OA group, there were several measures with significant negative interaction effects between single-task walking and balance score (worse balance, higher dlPFC activity) and Walk-12 score (lower self-perceived walking difficulty, higher dlPFC activity). For the PD group, there was an unexpected relationship to disease severity both in terms of Hoehn & Yahr group as well MDS-UPDRS score, with milder disease severity being associated with higher dlPFC activity during single-task walking. Tables of detailed results from the mixed-effects models for each hemoglobin type can be found in the supplementary material (supplementary Table S2-S6).

## 4 Discussion

This validation study tested hypotheses regarding convergent validity, discriminant validity and known-group validity, using a combined CBSI hemoglobin measure of dlPFC activity related to gait variables, self-reported questionnaires and clinical assessments. All statistically significant effects, where expected, were in line with our pre-defined hypotheses. However, many effects were non-significant. In the YA group, all three relationships were in line with hypotheses: a significant increase in dlPFC activity from single to dual-task walking, a positive relationship between dlPFC activity during dual-task walking and dual-task cost, and no relationship to anxiety score. In the OA group, only one out of seven tested hypotheses had a significant effect, an increase in dlPFC activity during single-task walking compared to rest. In the PD group, three out of nine hypothesized effects were significant: an increase in dlPFC activity during single-task walking, and a significant interaction effect with walking speed and step time variability.

### 4.1 Validity and known group assumptions

Effects in line with validation hypotheses belonged to both convergent and discriminant validity. For known group validity, no tested known group relationships were significant. This might reflect uncertainty in the literature about these effects. However, where there is uncertainty and less detailed theories about how these effects behave, an iterative validation process can supply information for refinement of these theories (de Vet et al., 2011). The known group assumption in hypothesis H2 that a higher disease severity would reflect in a higher dlPFC activity might have been incorrect. fNIRS studies on gait in PD (Al-Yahya et al., 2019, Dagan et al., 2021, Maidan et al., 2016, Maidan et al., 2015, Orcioli-Silva et al., 2020, Stuart et al., 2019, Vitorio et al., 2020) do not often report comparisons of disease severity groups or correlations to severity scores. However, a study by (Maidan et al., 2016) did find a higher prefrontal activity for participants with a lower (less severe) MDS-UPDRS motor score. The same pattern was found in this study in the exploratory analysis for HbO2, HbD and HbT. Another incorrect assumption could have been made in hypothesis H4, that the single-task to dual-task increase in dlPFC activity would be greater in OA and PD groups than YA. In fact, if the YA group experiences relatively little dlPFC activity in the single-task condition compared to the OA and PD groups, there might be more room for increase from single-task to dual-task. A lack of increase in PFC activity for PD from single to dual-task gait has been found in other studies (Dagan et al., 2021, Maidan et al., 2016, Orcioli-Silva et al., 2020, Vitorio et al., 2020). Interestingly, one study found that this changed to a significant increase when participants were in the OFF instead of ON state of medication (Dagan et al., 2021).

Given that all significant effects were in line with hypotheses and given similarities to other studies, we would argue that our measurements do reflect a valid measure of executive demand during single-task walking and dual-task walking. This is also supported due to our use of short-channel nuisance regressors, evaluation of signal quality, age-dependent modelling of differential path-length factor, and use of the AR-IRLS (Barker et al., 2013) model for suppressing possible physiology and movement artifacts.

### 4.2 Relationship between dlPFC activity and gait

We hypothesized that a higher prefrontal activity would be associated with a more “cognitively controlled” gait during single-task walking, with a lower walking speed and higher step time variability. This indeed seems to be the case at least in the PD group, although the interaction effects with gait variables were not significant in the OA group.

The reasons that dlPFC activity in the PD group is connected to gait variability and lower walking speed could be related to impairments specific to PD. In Fig. 3, it can be observed that the PD participants in general walked slower and had larger step time variability than the OA group. Gait slowness in PD is connected to hypokinesia and bradykinesia arising from dysfunction in the basal ganglia-thalamo-cortical system, affecting motor planning and scaling of motor output (Peterson and Horak, 2016). It has been observed that recruitment of additional brain areas seems to compensate for specific neurological impairments in PD (Peterson and Horak, 2016) and other neurological pathologies (Hamacher et al., 2015). The specific relation between dlPFC activity and slow gait speed could indicate such compensation. Moreover, such compensatory activity in PD is thought to require more voluntary control, resulting in increased gait variability (Peterson and Horak, 2016). The finding that dlPFC activity was connected to increased step time variability also supports such a theory.

For healthy older adults without neurological impairments, we did not find the same relationship between dlPFC activity and gait variables. Findings from other studies are mixed regarding this relationship, with significant associations between PFC activity and gait speed and variability during obstacle negotiation for OA (Mirelman et al., 2017) and PD (Maidan et al., 2016), but not during usual walking (Al-Yahya et al., 2019, Maidan et al., 2016, Mirelman et al., 2017). While step time variability has not been investigated as much, our exploratory analysis showed it to be one of the more consistent effects in terms of size and direction.

Many effects were non-significant. While main condition effects were well-powered, interaction effects between brain activity and covariates could have suffered from low power. It is known that fNIRS effect sizes can vary to a large extent depending on the task (Cockx et al., 2023), so it is possible that we failed to capture some more subtle effects in this study. There was also missing data for some covariates, which led to interaction effects involving those covariates having less participants than the other effects.

### 4.3 Exploratory analysis

Regarding the exploratory analysis, it is clear that analysis of oxygenated and deoxygenated hemoglobin can result in different outcomes, more for some effects (walking speed) and less for others (condition effects, step time variability). This could perhaps be due to the noise content or different susceptibility to motion artifacts depending on signal and effect of interest. Therefore, it is important to show both measures as recommended by best practices (Yücel et al., 2021) or use a combined measure (Hakim et al., 2022).

**Limitations**

Effects from the second level mixed models were used instead of correlations which are more typical for this type of validation study (Cooper et al., 2019), due to the ability of the second level models to use the covariance structure of the first level models to achieve better estimates (Santosa et al., 2018). However, correlation analysis of the CBSI GLM estimates to correlates led to largely the same results, the difference being a non-significant relationship for step time variability in the PD group.

While short-channel nuisance regressors were used in the analysis, it could have been of interest to use additional physiological signals like respiration. Systemic physiology augmented fNIRS (Scholkmann et al., 2022) could have enabled a more comprehensive understanding of the PFC activity during the walking tasks. This would also have been useful in understanding the effect of breathing (Scholkmann et al., 2013, Scholkmann and Wolf, 2013) during the auditory Stroop task.

Optodes were fitted on caps which were chosen according to the head size of participants, and reference points (e.g., nasion/inion) were controlled when fitting the cap. However, more precise spatial registration (Jaffe-Dax et al., 2020, Wyser et al., 2022) could likely have improved the precision with which underlying regions were measured.

## 5 Conclusion

We conclude that the results of this fNIRS study point towards the CBSI measure of dlPFC activity being a valid measure of executive demand during single-task walking and dual-task walking. However, there is still uncertainty about some relationships between gait performance and brain activity and the similarities and dissimilarities in different groups. An assumption of slow and variable gait being related to increased prefrontal activity might be too simple to be applied to disparate clinical groups. We found that a combined measure of oxygenated and deoxygenated hemoglobin reflected our validation hypotheses to the greatest extent, although reporting both measures can still provide important information. A detailed investigation of how performance of complex walking tasks relates to brain activity will be performed in future studies.

## CRediT authorship contribution statement

**Alexander Kvist:** Writing – review & editing, Writing – original draft, Visualization, Software, Methodology, Formal analysis, Data curation, Conceptualization. **Lucian Bezuidenhout:** Writing – review & editing, Methodology, Investigation, Conceptualization. **Hanna Johansson:** Writing – review & editing, Supervision, Methodology, Investigation, Conceptualization. **Franziska Albrecht:** Writing – review & editing, Supervision, Methodology. **David Moulaee Conradsson:** Writing – review & editing, Supervision, Methodology, Conceptualization. **Erika Franzén:** Writing – review & editing, Supervision, Project administration, Methodology, Investigation, Funding acquisition, Conceptualization.

## Declaration of Competing Interest

The authors declare that they have no known competing financial interests or personal relationships that could have appeared to influence the work reported in this paper.

## Data Availability

This study is preregistered on OSF, where additionally all scripts used for data analysis can be found (https://osf.io/uqy6d). The original data are not publicly available due to Swedish/EU law, but is located with restricted access in a central repository (Franzén, 2023). Data sharing will be regulated via a data transfer and user agreement upon a reasonable request.
