# Supplementary material for: Validation of fNIRS measurement of executive demand during walking with and without dual-task in younger and older adults and people with Parkinson’s disease
Source: Neuroimage Clin. 2024 Jul 1;43:103637. doi: 10.1016/j.nicl.2024.103637 (PMC11278929; doi:10.1016/j.nicl.2024.103637)
Supplement: Supplementary Data 1 [file mmc1.docx]

Table A1. fNIRS signal quality during each walking condition in terms of SCI and peak spectral power, averaged across all participants.

| Condition | SCI | Peak spectral power |
| --- | --- | --- |
| Standing auditory Stroop, mean (SD) | 0.967 (0.084) | 0.248 (0.141) |
| Single-task walking, mean (SD) | 0.968 (0.063) | 0.202 (0.119) |
| Dual-task walking, mean (SD) | 0.967 (0.061) | 0.194 (0.118) |
| Abbreviations: SD standard deviation, SCI scalp coupling index. | | |
